# Genetic Variants in Early-Onset Inflammatory Bowel Disease: Monogenic Causes and Clinical Implications

**DOI:** 10.3390/children12050536

**Published:** 2025-04-23

**Authors:** Duygu Demirtas Guner, Hacer Neslihan Bildik, Hulya Demir, Deniz Cagdas, Inci Nur Saltik Temizel, Riza Koksal Ozgul, Hayriye Hizarcioglu Gulsen, Cagman Tan, Begum Cicek, Hasan Ozen, Aysel Yuce, Ilhan Tezcan

**Affiliations:** 1 Department of Pediatrics, Division of Pediatric Gastroenterology, Hepatology and Nutrition, Hacettepe University, 06230 Ankara, Turkey; hudemir@hacettepe.edu.tr (H.D.); isaltik@hacettepe.edu.tr (I.N.S.T.); hayriyegulsen@hacettepe.edu.tr (H.H.G.); haozen@hacettepe.edu.tr (H.O.); ayuce@hacettepe.edu.tr (A.Y.); 2 Department of Immunology, Institute of Child Health, Pediatric Basic Sciences, Hacettepe University, 06230 Ankara, Turkey; hacernbildik@gmail.com (H.N.B.); ctan@erciyes.edu.tr (C.T.); begumozbek@hacettepe.edu.tr (B.C.); 3 Department of Pediatrics, Division of Pediatric Immunology, Hacettepe University, 06230 Ankara, Turkey; deniz.ayvaz@hacettepe.edu.tr (D.C.); fetezcan@hacettepe.edu.tr (I.T.); 4 Department of Pediatrics and Perinatal Pathological Sciences, Institute of Child Health, Hacettepe University, 06230 Ankara, Turkey; rkozgul@hacettepe.edu.tr

**Keywords:** Inflammatory bowel diseases, monogenic diseases, whole-exome sequencing, MEFV, NFKB2, SLC29A3

## Abstract

**Background/Objectives:** This study aims to identify genetic variants associated with early-onset inflammatory bowel disease (IBD) and to improve diagnostic and therapeutic approaches. In selected monogenic IBD cases, treatment included colchicine, interleukin-1 inhibitors, and hematopoietic stem cell transplantation. **Methods:** This study included patients with early-onset IBD, defined as IBD diagnosed before the age of 10, who were under follow-up at the Department of Pediatric Gastroenterology, Hacettepe University, and agreed to participate between December 2018 and April 2021. Whole-exome sequencing (WES) was performed prospectively in patients without a prior diagnosis of monogenic disease, while clinical and laboratory data were reviewed retrospectively. Identified variants were evaluated for pathogenicity using standard bioinformatics tools. **Results:** A total of 47 patients were enrolled, including 33 boys (70.2%) and 14 girls (29.8%). The median age at symptom onset was 36 months (IQR: 10–72), and the median age at diagnosis was 3.7 years (IQR: 1.5–7.6). Crohn’s disease was diagnosed in 53.2% (*n* = 25), ulcerative colitis in 38.3% (*n* = 18), and unclassified IBD in 8.5% (*n* = 4). Monogenic IBD was identified in 36.2% (*n* = 17) of patients, including nine with Familial Mediterranean Fever and others with glycogen storage disease type 1b (*n* = 2), XIAP deficiency, chronic granulomatous disease, DOCK8 deficiency, IL10 receptor alpha defect, LRBA deficiency, and NFKB2 deficiency (*n* = 1 each). A novel SLC29A3 gene variant (c.480_481delTGinsCA, p.V161I) (transcript ID: ENST00000479577.2) was identified in 76.6% (*n* = 36) of patients. **Conclusions:** This study underscores the importance of genetic variants in early-onset IBD, particularly MEFV and the novel NFKB2. The frequent detection of the SLC29A3 variant may suggest its potential involvement in the pathogenesis of the disease.

## 1. Introduction

Inflammatory bowel disease (IBD) is influenced by both genetic and environmental factors, with genetic predisposition playing a key role in early-onset cases. Although some of the responsible genes and rare variants associated with IBD have been identified, much remains to be discovered [1,2,3]. Infantile IBD, a subset of very early-onset IBD (VEO-IBD), refers to patients diagnosed with IBD before the age of 2 years, whereas VEO-IBD includes those diagnosed at or before six years of age. VEO-IBD is more likely than IBD presenting at older ages to be caused by monogenic disorders [4]. This reflects a high probability that a single genetic variant in a specific gene can lead to IBD, highlighting a critical need for genetic research in these early cases [3,5,6,7]. However, even in patients who present after the age of six, underlying monogenic causes may still be present [8,9,10].

Recent advancements in 2020 by the North American Society for Pediatric Gastroenterology, Hepatology and Nutrition (NASPGHAN) and in 2021 by the Pediatric IBD Porto Group of the European Society for Paediatric Gastroenterology, Hepatology and Nutrition (ESPGHAN) have identified numerous genes related to monogenic IBD. These findings highlight the evolving understanding of these complex genetic interactions and underscore the dynamic nature of genetic research in early-onset IBD. A growing number of these genes have been found to be involved in immune regulation (such as IL10, IL10RA, IL10RB, and FOXP3), microbial sensing and clearance (such as CYBB, CYBA, and NCF2), and epithelial barrier function (such as TTC7A), and they are now well-recognized in ESPGHAN and NASPGHAN recommendations [5,7].

Genetic investigation is essential for identifying monogenic causes associated with IBD. In order to increase the likelihood of detecting monogenic causes, our study included not only VEO-IBD patients but also those with early-onset IBD presenting before the age of 10. The primary aim of our study was to focus on monogenic causes or potential variants that may be associated with early-onset IBD. It was believed that detecting mutations in responsible genes or identifying potential variants could be crucial for the diagnosis, monitoring, and treatment of patients. Moreover, we focused on identifying and analyzing clinical and immunological features that could provide valuable insights into monogenic IBD.

## 2. Materials and Methods

### 2.1. Study Design and Setting

This study was conducted at the Department of Pediatric Gastroenterology, Hepatology, and Nutrition at the Hacettepe University Faculty of Medicine, a tertiary referral center in Türkiye. It included 47 patients with early-onset IBD, defined as IBD diagnosed before the age of 10, who were under follow-up at our department and agreed to participate between December 2018 and April 2021. The genetic component of the study was conducted prospectively, while clinical and laboratory data were collected retrospectively. Of the 47 patients, 45 had been diagnosed at our center, while two had received their diagnosis at other medical institutions but were regularly followed at our department.

### 2.2. Participants

Whole-exome sequencing (WES) was prospectively performed in 39 patients who had not previously been diagnosed with a monogenic disease. Prior to the study, some patients had undergone classical Sanger sequencing or targeted next-generation sequencing (IBD panels). In eight patients, these tests had already identified a monogenic cause of IBD and they were included in the study cohort. Therefore, only the remaining 39 patients underwent WES.

The diagnosis of IBD and its subtypes (Crohn’s disease, ulcerative colitis, and IBD-unclassified) was determined according to the revised Porto criteria established by the ESPGHAN Pediatric IBD Porto Group [11].

The inclusion criteria were early-onset IBD (diagnosed before the age of 10), regular follow-up at our department, and informed consent. Exclusion criteria included only refusal to participate.

### 2.3. Genetic Analysis

Identified variants were analyzed using clinical insight software and publicly accessible databases. The allelic frequencies of the identified nucleotide changes were compared to those in healthy individuals using the Exome Aggregation Consortium (ExAC) and Genome Aggregation Database (gnomAD) databases. To evaluate the pathogenicity of the nucleotide changes and their impact on the protein sequence, computer software programs such as PolyPhen, SIFT, and Mutation Taster were utilized [12,13].

The pathogenicity of the identified variants was assessed according to the standards and guidelines of the American College of Medical Genetics and Genomics (ACMG). Common variants that were consistently observed among the patients, regardless of their pathogenicity scores, were reported as findings, considering their potential involvement in the disease and as a foundation for future studies.

### 2.4. Clinical and Laboratory Data

Clinical and laboratory data were reviewed retrospectively from patient records. These included complete blood count, biochemistry, erythrocyte sedimentation rate (ESR), C-reactive protein (CRP), fecal calprotectin, immunoglobulin (Ig) levels (IgA, IgM, IgG, IgE), lymphocyte subsets, nitroblue tetrazolium (NBT) test, endoscopy, and endoscopic biopsy results. Not all tests were available for all patients.

### 2.5. Statistical Analysis

Statistical analyses were conducted using SPSS Windows, version 22.0. The normal distribution of numerical variables, which were provided with descriptive statistics, was assessed through visual methods (histograms and probability plots) and analytical methods (Kolmogorov–Smirnov/Shapiro–Wilk tests). If the numerical variables exhibited a normal distribution, they were presented as mean ± standard deviation. However, if the variables did not follow a normal distribution, median (25th–75th percentile) values were utilized. Categorical variables were presented as counts and percentages (%). The comparison of counts and percentages between groups was performed using the Chi-square test or Fisher’s exact test. A *p*-value of <0.05 was considered statistically significant.

### 2.6. Ethical Approval

Written consent was obtained from the parents for this study. The Institutional Ethics Committee approved the study (project number: GO 18/1188, decision number: GO 18/1188-20, decision date: 18 December 2018).

## 3. Results

### 3.1. Patient Characteristics and Clinical Presentation

A total of 88 pediatric IBD patients were identified at our center, of whom 53.4% had early-onset IBD and were included in the study. All patients had previously undergone upper and lower gastrointestinal endoscopic evaluations with histopathological sampling as part of their initial diagnostic work-up prior to their inclusion in the study. Based on these findings, 47 patients were diagnosed with early-onset IBD and were included in the study. The study cohort consisted of 47 patients: 14 girls (29.8%) and 33 boys (70.2%). The median ages of symptom onset and IBD diagnosis were 36 months (IQR: 10–72) and 3.7 years (IQR: 1.5–7.6). In this cohort, 53.2% (*n* = 25) were diagnosed with Crohn’s disease (CD), 38.3% (*n* = 18) with ulcerative colitis (UC), and 8.5% (*n* = 4) with unclassified inflammatory bowel disease (IBDU).

Among the 20 patients with infantile IBD, 9 (45%) were diagnosed with monogenic IBD, while 11 (31.4%) of the 35 patients with very early-onset IBD had monogenic IBD. In the infantile group, 60% (*n* = 12) of cases were diagnosed with CD, 20% (*n* = 4) with UC, and 20% (*n* = 4) with IBDU. For the VEO-IBD group, the distribution was 57.1% (*n* = 20) for CD, 31.4% (*n* = 11) for UC, and 11.4% (*n* = 4) for IBDU. Among all 47 patients, 20 (42.6%) were born to consanguineous parents, and 7 (14.9%) had a positive family history of IBD. Consanguinity was present in 11 of 17 patients with monogenic IBD (64.7%) and in 9 of 30 patients without monogenic IBD (30%) (*p* = 0.021). A family history of IBD was reported in two patients (11.8%) in the monogenic group and in five patients (16.7%) in the non-monogenic group.

At IBD presentation, 23 patients (48.9%) had bloody diarrhea, 14 (29.8%) had chronic diarrhea, and 13 (27.6%) reported abdominal pain. Weight loss and perianal abscess were observed in six patients each (12.8%). Furthermore, five patients (10.6%) had perianal fistula, five patients (10.6%) had fever, three patients (6.4%) had oral ulcers, three patients (6.4%) had symptoms of arthralgia or arthritis, and one patient (2.1%) had a perianal ulcer.

### 3.2. Laboratory and Immunologic Findings

The median ESR and CRP levels at IBD diagnosis were 38.5 mm/h (IQR:22–60.8, *n* = 40) and 2.91 mg/L (IQR: 0.61–7.6, *n* = 45), respectively. These values, based on available data, were provided to illustrate the overall inflammatory status at the time of diagnosis. No significant differences were observed between monogenic and non-monogenic IBD patients in these parameters. Immunoglobulin levels were assessed in most patients at diagnosis: IgA in 44 patients, IgM in 39, IgG in 40, and IgE in 39 patients. While the majority of values were within the normal range, a subset of patients showed variations. Specifically, IgA was low in six patients, two of whom had selective IgA deficiency. IgM was low in seven patients, IgG was low in eight, and IgE was low in two patients. Lymphocyte subsets were evaluated in 33 patients before the initiation of immunosuppressive treatment, showing sufficient levels in CD3 (94%), CD4 (90%), CD8 (88%), CD16–56 (92%), and CD19 (85%) lymphocytes. One patient was diagnosed with chronic granulomatous disease (CGD) following an abnormal NBT test, which led to the identification of a pathogenic CYBA mutation. This patient was not evaluated by WES, as the genetic diagnosis had already been established prior to study enrollment.

### 3.3. Genetic Variants Associated with Monogenic IBD

Of the 47 patients, 39 underwent WES, while the remaining eight patients had received prior diagnoses through classical Sanger sequencing and next-generation sequencing (NGS) for monogenic diseases. The variants detected in patients, along with their detailed classification as pathogenic, likely pathogenic, and variant of uncertain significance (VUS), likely benign, and benign, are presented in Table 1.

Monogenic IBD was identified in 17 patients (36.2%), with diagnoses including Familial Mediterranean Fever (FMF) (*n* = 9), glycogen storage disease type 1b (*n* = 2), XIAP deficiency (*n* = 1), CDG (*n* = 1), DOCK8 deficiency (*n* = 1), IL10 receptor alpha defect (*n* = 1), LRBA deficiency (*n* = 1), and NFKB2 deficiency (*n* = 1). Among these, one female patient with a heterozygous mutation in the NFKB2 gene was diagnosed with UC at age six, presenting with abdominal pain, intermittent blood in stool, selective IgA deficiency, and Coombs-negative hemolytic anemia. She developed a perianal abscess post-study.

### 3.4. Clinical Outcomes of Monogenic IBD Cases

A summary of the clinical characteristics of patients with monogenic IBD, including their age of symptom onset, presenting symptoms, IBD type, specific genetic diagnoses, and outcomes, is presented in Table 2. Table 3 provides a comparative overview of patients with and without monogenic IBD in terms of demographic and clinical features. Additionally, no statistically significant differences between the two groups were found in age at symptom onset, age at IBD diagnosis, or laboratory inflammatory markers (ESR, CRP, and fecal calprotectin) based on available data.

Hematopoietic stem cell transplantation (HSCT) was successfully performed in three patients diagnosed with IL10 receptor alpha defect, DOCK8 deficiency, and LRBA deficiency. One patient with XIAP deficiency died from sepsis during the donor investigation for HSCT.

### 3.5. Potential Genetic Variants in Early-Onset IBD

In all patients with variants in SLC29A3, NLRP6, and IL1RL1, the same specific variant was consistently identified in each respective gene. A variant in the SLC29A3 gene (c.480_481delTGinsCA, p.V161I) (transcript ID: ENST00000479577.2), which has not been previously associated with IBD, was detected in 36 patients (76.6%). According to Ensemble-Human (GRCh38.p14), the transcript with ENST00000479577.2 ID of the SLC29A3 gene has valine at position 161. For this variant, 27 patients were homozygous, 8 were heterozygous, and 1 was compound heterozygous.

In the NLRP6 gene, a variant (c.1082_1083delATinsTC, p.Y361F) (transcript ID: ENST00000534750.6) was identified in 35 patients (74.5%), with 17 patients homozygous and 18 heterozygous.

A variant in the IL1RL1 gene (c.1501_1502delCAinsAG, p.Q501R) (transcript ID: ENST00000233954.6) was found in 21 patients (44.7%), 1 homozygous and 20 heterozygous.

### 3.6. Genetic Variants and Their Impact on Treatment

Thirteen patients received biologic agents for IBD management. These included tumor necrosis factor-alpha (TNF-α) inhibitors such as infliximab and adalimumab, as well as interleukin-1 (IL-1) blockers such as anakinra (an interleukin-1 receptor antagonist) and canakinumab (a monoclonal antibody targeting interleukin-1β). The choice of agent varied based on clinical presentation and the underlying genetic diagnosis, particularly in patients with autoinflammatory or immune dysregulation syndromes. A variant in the SLC29A3 gene was detected in all 13 patients (100%) receiving biologics, and in 23 of 34 patients (67.6%) not receiving biologics. The frequency of the SLC29A3 variant was significantly higher in patients receiving biologic agents compared to those not receiving biologic agents (*p* = 0.021). Similarly, MEFV gene variants were more common in patients receiving biological agents (*n* = 10/13, 76.3%) than in those not receiving biological agents (*n* = 15/34, 44.1%), with a statistically significant difference (*p* = 0.044). These findings suggest that the presence of certain genetic variants, particularly in the MEFV and SLC29A3 genes, may play a role in determining the need for biologic agents in patients with early-onset IBD.

In contrast, biologic agents such as anakinra and canakinumab, which are primarily used in FMF and not routinely used in IBD, have been used to treat FMF in patients with an IBD diagnosis.

Hematopoietic stem cell transplantation is an important treatment strategy for some patients with monogenic IBD. In patients with IL10 receptor deficiency, DOCK8 deficiency, and LRBA deficiency, HSCT resulted in the remission of IBD and discontinuation of treatment.

These findings suggest that certain genetic variants, particularly FMF and IL10 receptor deficiency, significantly influence treatment strategies, highlighting the importance of genetic disorders in the selection of biologic therapies and HSCT approaches.

## 4. Discussion

### 4.1. Monogenic IBD: Regional and Novel Genetic Perspectives

The primary aim of our study was to investigate the responsible genes associated with early-onset IBD. A total of 17 patients (36.2%) were diagnosed with monogenic IBD, with MEFV being the most common gene, followed by SLC37A4, CYBA, XIAP, DOCK8, IL10RA, LRBA, and NFKB2. NFKB2 has not been previously associated with monogenic IBD in NASPGHAN and ESPGHAN articles [5,7]. However, genome-wide association studies (GWAS) suggested its potential role in IBD susceptibility [14]. A prior study identified NFKB2 mutations in a child with refractory diarrhea [15]. Similarly, our study identified a female patient with a heterozygous mutation in the NFKB2 gene who was diagnosed with UC, presenting a complex clinical picture involving multiple systemic issues. These findings suggest that NFKB2 mutations could represent a novel genetic marker for susceptibility to early-onset IBD, warranting further investigation in larger cohorts.

In contrast with studies from North America and Iran, where IL10RA/B and MVK are common [16,17], our study identified MEFV as the predominant variant. This highlights the importance of region-specific genetic analyses.

In the literature, monogenic causes have been identified in between 15% and 20% of VEO-IBD cases, with a range from 0% to 33% [5,7]. A study of 1005 pediatric IBD patients using WES reported monogenic causes in 3% of cases, with symptom onset before age two being significantly associated with monogenic IBD [18]. Another study using NGS on 62 patients diagnosed with infantile-onset IBD revealed an underlying monogenic cause in 31% of the patients. This study also found an association between monogenic IBD, parental consanguinity, and symptom onset before the age of six months [9].

In our study of 47 early-onset IBD patients, monogenic causes were identified in 36.2%, with no significant difference in symptom onset or diagnosis age between monogenic and non-monogenic groups.

### 4.2. Parental Consanguinity and Its Implications in Monogenic IBD

Similar to previous reports, our study found an association between parental consanguinity and monogenic IBD. The rate of parental consanguinity was 64.7% in patients with monogenic IBD and 30% in those without, a difference that was statistically significant (*p* = 0.021). Therefore, it is important to consider the possibility of an underlying monogenic cause, particularly in early-onset IBD, especially in cases where there is parental consanguinity.

While a positive family history of IBD is common in both monogenic and non-monogenic IBD patients, it does not necessarily indicate a specific association with monogenic causes (5). In our study, 11.8% of patients with monogenic IBD and 16.7% of patients without monogenic IBD had a family history of IBD, suggesting that family history alone is not a reliable indicator of monogenic IBD.

### 4.3. The Role of FMF and MEFV Variants in Early-Onset IBD

The most commonly identified monogenic cause in our study was FMF (19.1%). Familial Mediterranean Fever is an autoinflammatory disease characterized by recurrent fever, serositis, inflammation, and a predisposition to vasculitis and IBD [19]. The prevalence of MEFV gene variants was 76.3% in patients receiving biological agents for IBD, and 44.1% in patients not receiving them.

In a previous study at our hospital, MEFV mutations were detected in 25.7% of children diagnosed with IBD, and FMF was diagnosed in 21.2% of the cohort, which is higher than the prevalence of FMF in the general population (0.1%) [20]. In our current study, the prevalence of MEFV variants was found to be 53.2%, while the FMF diagnosis rate was 19.1%. Although the FMF diagnosis rate was similar to previous findings, the higher prevalence of MEFV variants suggests that MEFV mutations may contribute to early-onset intestinal inflammation and increased susceptibility to IBD.

In another study conducted in our country in 2013, 12 out of 78 FMF patients (15.4%) were diagnosed with IBD. The median age at FMF diagnosis was 7.5 ± 3.4 years. This finding supports previous evidence that FMF patients have a higher incidence of IBD compared to the general population, suggesting that MEFV variants may increase susceptibility to IBD [21]. Considering the high prevalence of FMF in our country, it is important to evaluate MEFV variants in patients diagnosed with IBD, especially in early-onset IBD. In line with the literature, our study also revealed that CD was more commonly observed in patients diagnosed with FMF, with a prevalence of 55.6% [20,21,22].

### 4.4. SLC29A3 Variants and Their Potential Role in IBD

The SLC29A3 gene variant (c.480_481delTGinsCA, p.V161I) was identified in 76.6% of our patients. According to the 1000 Genomes Project, this variant has a minor allele frequency (MAF) of 0% and is classified as a VUS based on ACMG criteria [23].

Mutations in the SLC29A3 gene have been linked to H syndrome and pigmented hypertrichosis with insulin-dependent diabetes mellitus (PHID), both autosomal recessive disorders [24,25,26]. Although these conditions are distinct from IBD, the expression of SLC29A3 in the gastrointestinal system during mouse embryogenesis may suggest its potential involvement in IBD [26]. The frequent detection of this variant in our cohort further supports the hypothesis that SLC29A3 may contribute to early-onset IBD, though additional studies are needed to confirm this association.

In our study, the SLC29A3 variant was present in 100% of patients receiving biologic agents for IBD compared to 67.6% of those not receiving biologics. Additionally, the coexistence of SLC29A3 and MEFV variants was significantly more frequent in patients on biologic therapy. These findings suggest that these variants may be associated with a more severe form of IBD that is less responsive to conventional treatments and may require the use of biologics.

### 4.5. NLRP6 and IL1RL1 Variants and Their Potential Role in IBD

The variant identified in the NLRP6 gene (c.1082_1083delATinsTC, p.Y361F) has a MAF of 0.79% according to the 1000 Genomes Project [22] and is classified as a VUS according to ACMG classification [23]. However, based on these databases, the variant is considered to have potential pathogenicity.

Studies indicate that NLRP6 plays a role in regulating the intestinal microbiota and maintaining an antibacterial and anti-inflammatory balance in the gastrointestinal system [27,28]. Additionally, NLRP6 deficiency has been linked to intestinal inflammation, hyperplasia, and worsened colitis in mice, suggesting that disruptions in the inflammasome pathway may increase susceptibility to IBD [29]. The detection of this variant in the majority of our patients suggests a potential role in early-onset IBD.

The IL1RL1 variant (c.1501_1502delCAinsAG, p.Q501R) has a MAF of 0.35% according to the 1000 Genomes Project [23]. Although classified as likely benign by the ACMG, the same variant was observed in all 21 patients in whom an IL1RL1 variant was detected. Previous studies have linked IL1RL1 variants to IBD, with evidence suggesting an association between common IL1RL1 polymorphisms and an increased risk of CD and UC [30]. Therefore, the IL1RL1 variant identified in our cohort may contribute to early-onset IBD.

In summary, SLC29A3 (76.6%), IL1RL1 (44.7%), and NLRP6 (74.5%) variants were identified in a significant portion of our IBD cohort, but also in healthy individuals (according to gnomAD).

These findings suggest that these variants may trigger inflammatory mechanisms rather than serving as monogenic causes. We hypothesize that variants (polymorphisms) in these genes contribute to IBD progression and may coexist in the same patient. Larger cohorts and functional studies are needed to confirm their role in disease progression.

The limitations of our study include the inability to perform family segregation analyses due to limited financial resources, the absence of a control group, and the lack of functional studies to validate the identified variants. The significance of these variants requires further clarification through functional studies. Additionally, clinical and laboratory data were collected retrospectively from patient records, which may have resulted in incomplete or missing data for certain variables.

### 4.6. Treatment Implications in Monogenic IBD

The identification of monogenic forms of IBD has significant therapeutic implications, as management strategies may differ substantially from those used in polygenic or idiopathic IBD. In our cohort, treatment regimens included conventional therapies such as corticosteroids, mesalazine, and immunomodulators, as well as biologic agents and, in selective cases, HSCT. Hematopoietic stem cell transplantation has been reported as curative for specific monogenic IBD disorders, including CGD, IPEX syndrome, and IL10RA/B deficiencies [31]. The increasing recognition of underlying inborn errors of immunity in monogenic IBD highlights the importance of early and accurate molecular diagnosis, which can help guide personalized therapeutic approaches [32].

## 5. Conclusions

This study underscores the importance of evaluating genetic variants, particularly MEFV, in early-onset IBD and identifies NFKB2 as a potential novel monogenic cause. Although SLC29A3 variants were frequently observed in this cohort, further functional studies are essential to elucidate their role in the pathogenesis of early-onset IBD. In conclusion, monogenic causes have been identified in a significant proportion of patients with early-onset IBD, highlighting the importance of genetic evaluation in this population. These findings also underscore the clinical value of genetic testing in guiding treatment decisions, including the use of targeted therapies or HSCT in selected patients.

## Figures and Tables

**Table 1:** Gene variants.

Gene	c. DNA	Protein	Inheritance	Patient Number (Percent)	ACMG
*SLC29A3*	c.480_481delTGinsCA	p.V161I	AR	36 (76.6%)	VUS
*NLRP6*	c.1082_1083delATinsTC	p.Y361F	-	35 (74.5%)	VUS
*MEFV*	c.2080A > G	p.M694V	AR	25 (53.2%)	Pathogenic
	c.2040G > C	p.M680I			Pathogenic
*IL1RL1*	c.1501_1502delCAinsAG	p.Q501R	-	21 (44.7%)	VUS
*DUOX2*	c.4298T > A	p.I1433N	AR	7 (14.9%)	VUS
	c.4301_4302insGCCAGTGGTTGGCTGGCTCCACCA	p.Q1434_E1435insPVVGWLHQ			VUS
	c.462G > A	p.R154R			Likely Benign
	c.1621C > T	p.R541W			VUS
	c.2182G > A	p.A728T			Benign
	c.3042G > A	p.A1014A			Benign
	c.1060C > T	p.R354W			VUS
	c.4485C > T	p.F1495F			Likely Benign
*IL10RA*	c.G477A	p.Trp.159X	AR	6 (12.8%)	Pathogenic
	c.781C > T	p.R261W			VUS
	c.499T > C	p.Tyr167His, chr11:117993372			VUS
	c.884C > T	p.P295L			Likely Benign
	c.330C > G	p.N110K			VUS
	c.499T > C	p.Y167H			VUS
*SLC9A3*	c.1954A > G	p.I652V	AR	5 (10.6%)	VUS
	c.412G > A	p.G138S			VUS
	c.1471A > G	p.I491V			VUS
	c.2475-7C > T				Likely Benign
*FCGR2A*	c.184_185delCAinsTG	p.Q62W	AR/AD	4 (8.5%)	VUS
*MYO5B*	c.5094_5095delCTinsGC	p.L1698_L1699delinsLL	AR	3 (6.4%)	Likely Benign
	c.5108T > C	p.V1703A			Likely Benign
	c.2645G > A	p.R882Q			VUS
*NOX1*	c.967G > A	p.D323N	-	3 (6.4%)	Benign
	c.109G > A	p.D37N			VUS
	c.749G > A	p.R250Q			VUS
*NOD2*	c.3019dupC	p.L1007fs*2	-	2 (4.3%)	VUS
	c.160G > A	p.E54K			VUS
	c.2051G > A	p.R684Q			Likely Benign
*SLC37A4*	c.1042_1043delCT	p.Leu348fs*53	AR	2 (4.3%)	Pathogenic
	c.1015G > T	p.Gly339Cys			Pathogenic
*SLC26A3*	c.405G > A	p.M135I	AR	2 (4.3%)	VUS
	c.295G > A	p.D99N			VUS
*STXBP3*	c.635A > T	p.E212V	-	2 (4.3%)	VUS
	c.1373C > T	p.P458L			VUS
*TRIM22*	c.962G > A	p.R321K	-	2 (4.3%)	Benign
	c.774G > A	p.R258R			VUS
*CYBA*	c.74G > A	p.Gly25Asp	AR	1 (2.1%)	Likely Pathogenic
*DOCK8*			AR	1 (2.1%)	
*IKZF2*	c.971T > G	p.V324G	-	1 (2.1%)	VUS
*IL33*	c.154A > G	p.M52V	-	1 (2.1%)	VUS
*IRAK1*	c.1106G > A	p.G369E	-	1 (2.1%)	VUS
*LIG1*	c.928G > A	p.D310N	AR	1 (2.1%)	VUS
*LRBA*			AR	1 (2.1%)	
*NFAT5*	c.152A > C	p.K51T	-	1 (2.1%)	VUS
*NFKB2*	c.1832G > A	p.Arg611Gln	AD	1 (2.1%)	VUS
*NLRP2*	c.11C > T	p.S4L	-	1 (2.1%)	Benign
*NLRP12*	c.79_81delAAG	p.K27del	AD	1 (2.1%)	VUS
*RNF186*	c.151C > T	p.R51W	-	1 (2.1%)	VUS
*TMPRSS6*	c.1842-3_1842-2delCA		AR	1 (2.1%)	Likely Pathogenic
	c.1842-7A > C				Likely Benign
*TRAF3*	c.763delC	p.R255fs*13	-	1 (2.1%)	Likely Pathogenic
*XIAP*	c.518G > A	p.Trp173Ter	XR	1 (2.1%)	Likely Pathogenic
*ZNF300*	c.604G > A	p.V202I	-	1 (2.1%)	VUS

ACMG: The American College of Medical Genetics and Genomics, AD: Autosomal dominant, AR: Autosomal recessive, XR: X-linked recessive, VUS: Variant of uncertain significance.

**Table 2:** Clinical characteristics of patients with monogenic IBD.

Patient ID	Sex	IBD Type	Age of Symptom Onset (Months)	Age at IBD Diagnosis (Years)	Symptoms and/or Findings	Diagnosis	Outcome
Patient 1	M	CD	1	1	Fever, bloody diarrhea, perianal abscess, perianal fistula	XIAP deficiency	ADA planned but not given due to HLH + AZT + mesalazine + partial enteral nutrition + ileostomy + died before HSCT
Patient 2	M	CD	4	1	Bloody diarrhea, perianal abscess, perianal fistula	IL10RA	HSCT performed + no IBD treatment
Patient 3	M	IBDU	18	5	Diarrhea	CGD	Steroid + interferon-γ + TMP-SMX prophylaxis + donor investigation for HSCT (previosly on mesalazine)
Patient 4	M	CD	18	2	Diarrhea, hepatosplenomegaly	GSD 1b	AZT + mesalazine + G-CSF
Patient 5	M	CD	72	6	Diarrhea, hepatosplenomegaly, oral aphthous ulcers, perianal ulcer	GSD 1b	AZT + mesalazine + G-CSF
Patient 6	M	UC	84	7	Abdominal pain	DOCK8 deficiency	HSCT performed + no IBD treatment + treatment for sclerosing cholangitis (corticosteroid taper + monthly IVIG + UDCA + propranolol)
Patient 7	M	CD	114	10	Diarrhea, hepatosplenomegaly	LRBA deficiency	HSCT performed + no IBD treatment
Patient 8	F	UC	66	6	Abdominal pain, blood in stool, hepatosplenomegaly, growth retardation	NFKB2 deficiency	Mesalazine (oral and rectal) + cyclosporine + IVIG (previously on steroids and AZT)
Patient 9	M	IBDU	2	0.5	Bloody diarrhea, perianal abscess	FMF	Colchicine
Patient 10	M	CD	10	1	Perianal abscess, perianal fistula	FMF	AZT + colchicine
Patient 11	F	UC	2	1	Bloody diarrhea	FMF	Colchicine + canakinumab
Patient 12	F	UC	10	2	Diarrhea, growth retardation, perianal fistula, with colostomy (due to cecal perforation)	FMF	Mesalazine (oral and enema) + colchicine
Patient 13	M	CD	0	6	Diarrhea	FMF	ADA + AZT + mesalazine + colchicine
Patient 14	F	CD	48	7	Abdominal pain	FMF	Anakinra + mesalazine
Patient 15	M	CD	90	8	Abdominal pain, diarrhea	FMF	Mesalazine + colchicine
Patient 16	F	CD	108	9	Abdominal pain	FMF	Colchicine + canakinumab + mesalazine (previously on infliximab)
Patient 17	F	UC	96	9	Bloody diarrhea, oral aphthous ulcers	FMF	Post-colectomy + colchicine

ADA: Adalimumab, AZT: Azathioprine, CD: Crohn’s disease, CGD: Chronic granulomatous disease, F: Female, FMF: Familial Mediterranean Fever, G-CSF: Granulocyte colony-stimulating factor, GSD 1b: Glycogen storage disease type 1b, HLH: Hemophagocytic lymphohistiocytosis, HSCT: Hematopoietic stem cell transplantation, IBDU: Unclassified inflammatory bowel disease, IFX: Infliximab, IL10RA: IL10 receptor alpha defect, IVIG: Intravenous immunoglobulin, M: Male, MMF: Mycophenolate mofetil, TMP-SMX: Trimethoprim-sulfamethoxazole, UC: Ulcerative colitis, UDCA: Ursodeoxycholic acid.

**Table 3:** Comparison between monogenic and non-monogenic IBD patients.

Characteristic	Monogenic IBD (*n* = 17)	Non-Monogenic IBD (*n* = 30)	*p* Value
Female, n (%)	6 (35.3%)	8 (26.7%)	*p* = 0.53
Male, n (%)	11 (64.7%)	22 (73.3%)	*p* = 0.53
Crohn’s disease, n (%)	10 (58.8%)	15 (50%)	*p* = 0.56
Ulcerative colitis, n (%)	5 (29.4%)	13 (43.3%)	*p* = 0.35
IBDU, n (%)	2 (11.8%)	2 (6.7%)	*p* = 0.61
Infantile IBD, n (%)	9 (52.9%)	11 (36.7%)	*p* = 0.28
VEO-IBD, n (%)	11 (64.7%)	24 (80%)	*p* = 0.31
Mean age, years (± SD)	6.7 ± 4.7	8.2 ± 4	*p* = 0.26
Median age at symptom onset, months (IQR)	18 (3–87)	36 (11.5–72)	*p* = 0.70
Median age at IBD diagnosis, years (IQR)	6.1 (1.2–7.8)	3.6 (1.5–6.3)	*p* = 0.74
Perianal abscess, n (%)	4 (23.5%)	2 (6.7%)	*p* = 0.17
Perianal fistula, n (%)	4 (23.5%)	1 (3.3%)	*p* = 0.051
Perianal ulcer, n (%)	1 (5.9%)	0	*p* = 0.36
Family history of IBD, n (%)	2 (11.8%)	5 (16.7%)	*p* = 1
Consanguinity in parents, n (%)	11 (64.7%)	9 (30%)	*p* = 0.021

## Data Availability

The original contributions presented in this study are included in the article. Further inquiries can be directed to the corresponding author.
